# Comparison and correlation of candidal colonization in diabetic patients and normal individuals

**DOI:** 10.1186/2251-6581-13-66

**Published:** 2014-06-04

**Authors:** Bakthavatchalam Pallavan, Venkatapathy Ramesh, Balamurali Pennagaram Dhanasekaran, Nirima Oza, Sudip indu, Vasupradha Govindarajan

**Affiliations:** 1 Department of oral pathology and microbiology, Mahatma Gandhi post graduate institute of dental sciences, Pondicherry, India

**Keywords:** Exfoliative cytology, Diabetic patients, Candida albicans, Colonization, Candidiasis

## Abstract

**Background:**

Diabetes mellitus is a common universal endocrine disorder with decreased host immunity towards infections. In these people the most common opportunistic infection is oral candidiasis. Oral candidiasis is most commonly caused by yeast like fungus *Candida albicans*. In healthy individuals these microorganisms are believed to be commensals but in diabetic patients, it forms severe colonization, even in the absence of any clinically evident oral candidiasis. This type of subclinical colonization can make them more prone to develop deeper mucosal colonization with further dissemination via blood. The aim of this study is to compare the frequency and severity of oral candidal colonization in diabetic patients with normal individuals through cytological method.

**Methods:**

30 cases of diabetic patients and 30 cases of normal healthy individuals were examined to determine the oral candidal colonization through oral exfoliative cytological methods. Statistical analysis was done using the Chi - square test.

**Results:**

A statistically significant increase in the candidal colonization was observed in diabetic patients as compared to normal individuals.

**Conclusions:**

Oral exfoliative cytological method is an easy and effective chair side technique to assess the oral candidal colonization in the diabetic group.

## Background

Oral cavity harbours various normal microbial flora among these *Candida* is a harmless commensal organism present in 20 to 40% of healthy individuals [1]. *Candida* is an opportunistic yeast-like fungus among these *Candida albicans* is the most common species causing oral candidiasis [2,3]. Several local and systemic factors of diabetes mellitus can predispose oral candidiasis.

Oral lesions are prevalent among diabetics and out of these lesions; oral candidiasis is the most common which can be diagnosed by their differential patterns of mucosal changes like erythematous, pseudomembranous and curd like plaques [4,5]. However, in diabetic group, *Candida albicans* colonization can occur even in the absence of any clinical lesion.

Candidal colonization can be confirmed by culture methods which are very time consuming on the other hand oral exfoliative cytology is a very easy and effective chair side method.

The purpose of this study was to assess the frequency of candidal colonization in diabetics.

## Methods

A case control study was carried out in which samples were selected in a simple random sampling technique which included 30 cases of diabetics and 30 cases of age and gender matched controls (normal individuals). Informed consent from each person was taken and ethical clearance has been obtained for this study. A detailed history from the patient was taken which includes age, sex and duration of diabetes and the blood sugar level. The age groups of the samples were within 40 – 60 years. A thorough intraoral examination was carried out for all patients. People were excluded from the study with the following factors:

Patients with clinical lesions of oral candidiasis

Patients wearing dentures

Edentulous patients

Patients with harmful oral habits

Recent history of antibiotic therapy

Acute and chronic diseases

Endocrine disorders

Immunodeficiency diseases

Nutritional deficiency diseases

Exfoliative oral cytology was carried out for all the 60 cases. Mucosal scrapings were taken from the buccal mucosa with the help of wet wooden ice cream spatula. These scrapings were made into smears on a glass microscope slides with circular motion. Prepared smears were fixed immediately with 95% ethyl alcohol in a coupling jar.

Fixed smears were stained with PAS (Periodic Acid Schiff) reagent. McManus PAS method was used as a staining technique to identify the fungus in oral cytological smears [6]. This method is described as follows:

1. Progressively hydrate the cytological smear with decreasing concentration of alcohol to distilled water

2. Oxidize in periodic acid for 5 minutes

3. Rinse well in distilled water

4. Place in Schiff reagent for 15 minutes

5. Wash in running tap water for 10 minutes to allow pink colour to develop.

6. Counterstain with 0.2% working light green solution for a few seconds

7. Dehydrate in 95% alcohol and absolute alcohol

8. Clear in xylene

9. Mount with DPX

### Quality control

Peripheral blood smears have been used as a positive control in standardizing the PAS reagent staining technique. The PAS reagent selectively stains the granules of leukocytes.

All the stained slides were examined for *Candida* in a binocular research microscope under high power magnification (40×). These are seen as magenta to red colour structures in a pale green background.

## Results

In the present case control study, the aim was to compare the frequency and severity of oral candidal colonization in diabetics with normal individuals through oral exfoliative cytological method. Microscopic analysis of stained slide was done for all 60 cases which included 30 normal individuals and 30 diabetics.

Various morphological forms of *Candida* were observed under light microscopy which includes,

• Yeast (Figure 1)

• Hyphae (Figure 2)

• True hyphae (Figure 3)

• Budding yeast (Figure 4)

• Pseudohyphae (Figure 5)

• Pseudohyphae with budding yeast (Figure 6)

• Branching hyphae with budding yeast (Figure 7)

• Germ tube (Figure 8)

**Figure 1:**
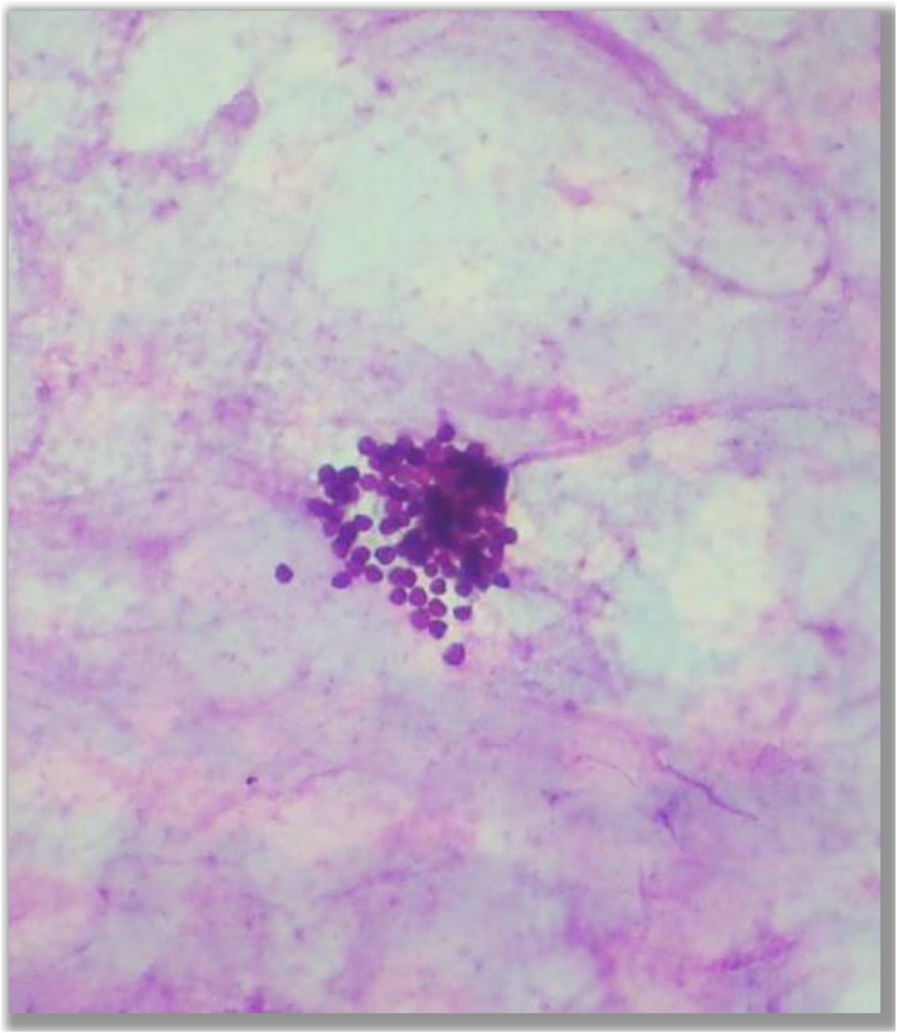
**Yeast.** Photo micrograph showing yeast form of fungus in the PAS stained cytological smears (400×).

**Figure 2:**
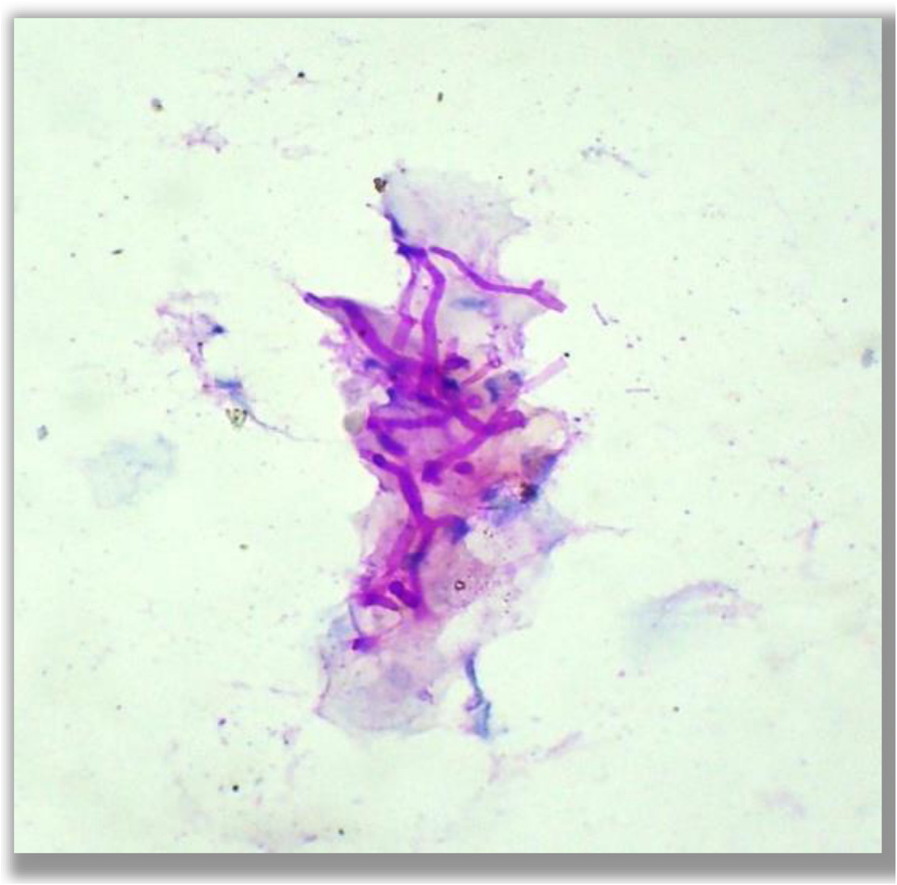
**Hyphae.** Photo micrograph showing hyphae form of fungus in the PAS stained cytological smears (400×).

**Figure 3:**
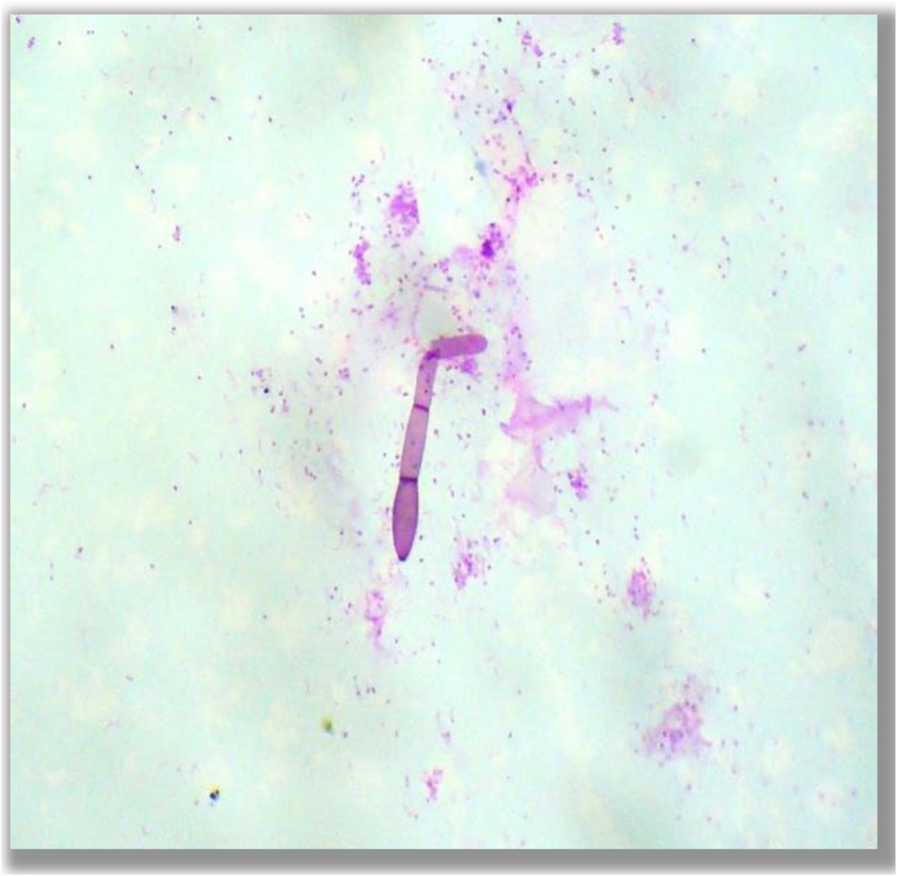
**True hyphae.** Photomicrograph showing true hyphae form of fungus in the PAS stained cytological smears (400×).

**Figure 4:**
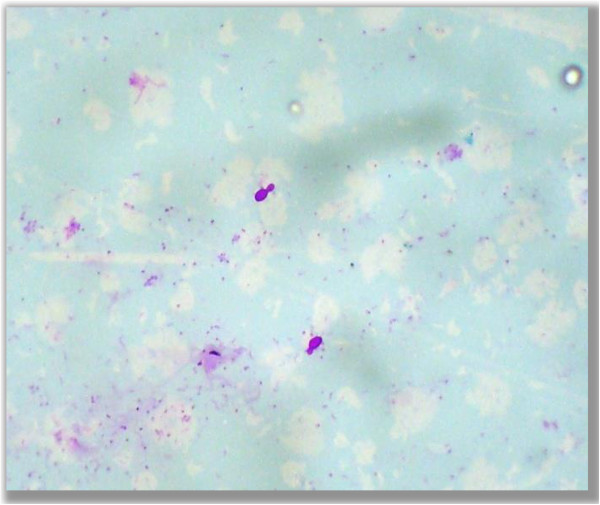
**Budding yeast.** Photomicrograph showing budding yeast form of fungus in the PAS stained cytological smears (400×).

**Figure 5:**
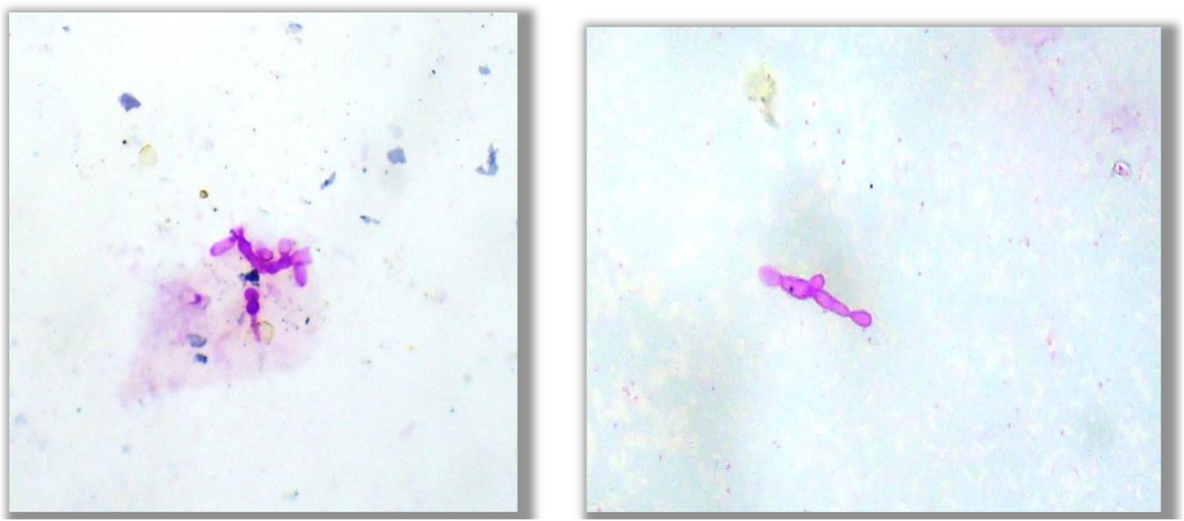
**Pseudo hyphae.** Photomicrographs showing pseudo hyphae form of fungus in the PAS stained cytological smear (400×).

**Figure 6:**
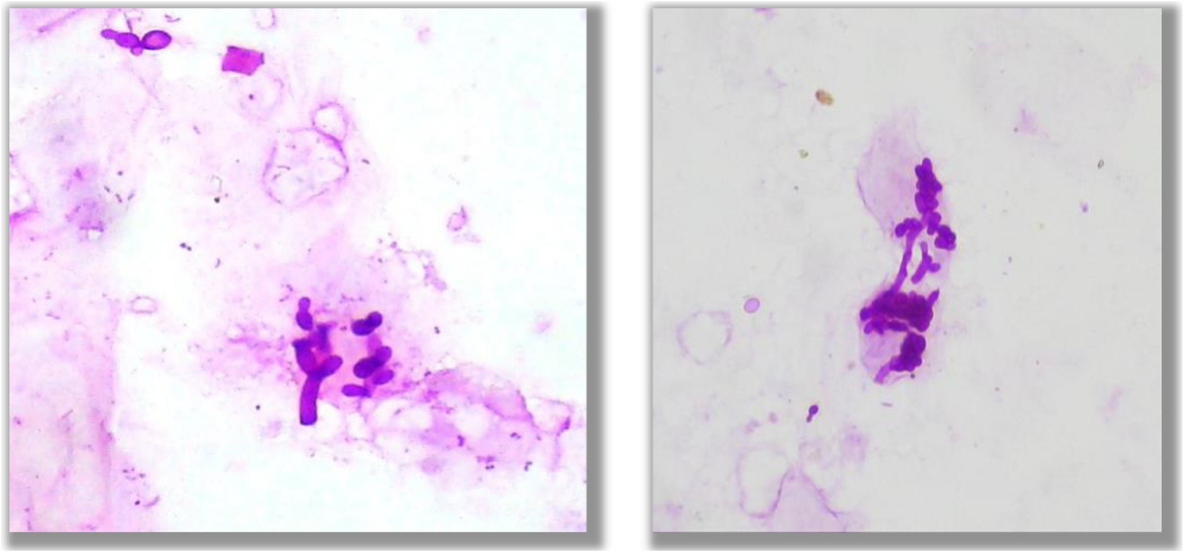
**Pseudo hyphae with budding yeast.** Photomicrographs showing pseudo hyphae with budding yeast form of fungus in the PAS stained cytological smear (400×).

**Figure 7:**
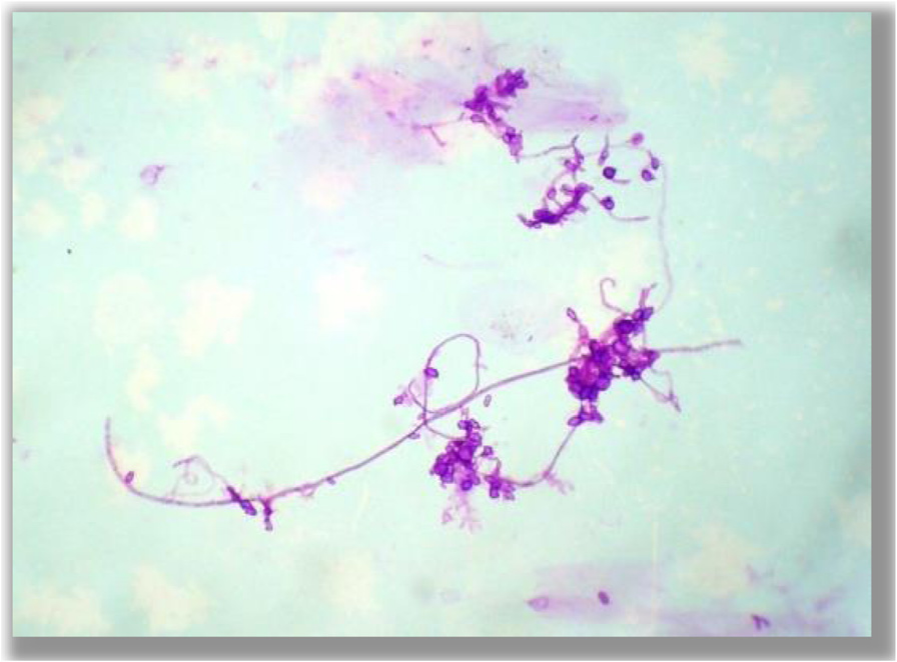
**Branching hyphae with budding yeast.** Photomicrograph showing branching hyphae with budding yeast form of fungus in PAS stained the cytological smear (400×).

**Figure 8:**
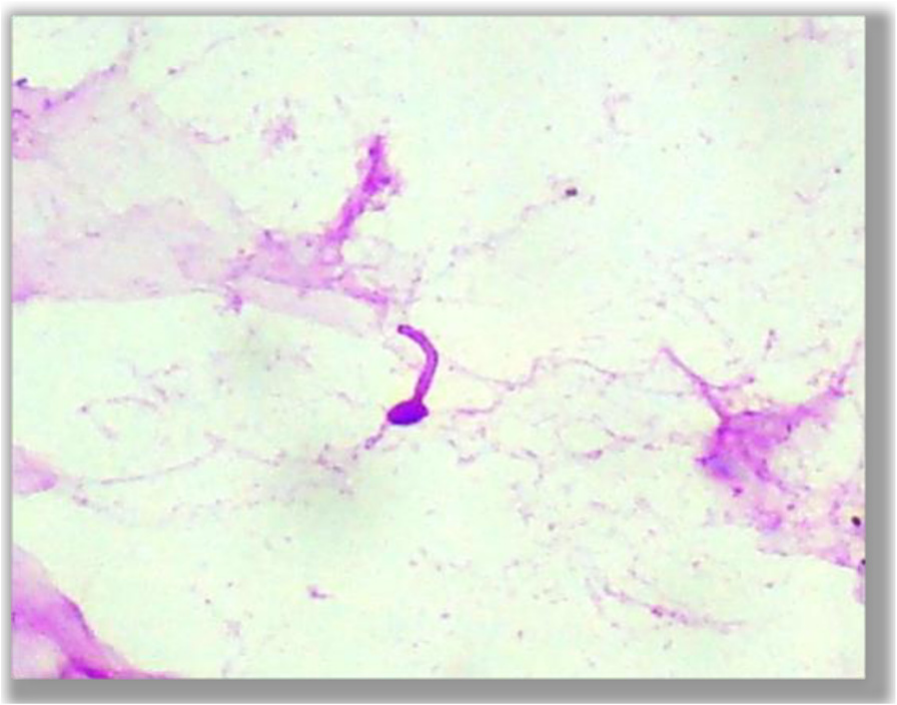
**Germ tube.** Photomicrograph showing a germ tube form of fungus in the PAS stained cytological smear (400×).

Based on the morphological forms of *Candida*, scoring criteria [7] was made (Table 1). Based on scoring values, grades of candidal colonization were also made (Table 2).

**Table 1:** **Scoring criteria for *candidal *** **colonization**

**Score**	**Inference**
0	No evidence of Candida
1	Presence of yeast and hyphae without budding yeast
2	Many numbers of yeast, hyphae with budding yeast
3	Many numbers of yeast, hyphae with pseudo hyphae < 3 in number
4	Many numbers of yeast, hyphae with pseudo hyphae > 3 in number& branching mycelium

**Table 2:** **Grades of *candidal *** **colonization**

**Value**	**Grades**
0	No colonization
1	Mild
2 & 3	Moderate
4	Severe

Severe candidal colonization was seen in 43.3% of diabetic patients and 10% of normal individuals. But in both normal individuals and diabetic patients, 6% of people had moderate colonization. However, 70% of normal individuals and just 11% of diabetic patients had mild colonization (Table 3).

**Table 3:** **Comparison of *candidal *** **colonization between diabetics and normal individuals**

**Candidal colonisation**	**Diabetics N (%)**	**Normal individuals N (%)**
**Mild**	11 (36.7)	21 (70)
**Moderate**	6 (20)	6 (20)
**Severe**	13 (43.3)	3 (10)
**Total**	30 (100)	30 (100)

Under Chi-square test statistical analysis, it was found that differences in the distribution of candidal colonization between diabetic patients and normal individuals were statistically significant (p value = 0.009). These findings were similar to that of Gary A. Bartholomew et al [8] where the severity of candidal colonization was greater in diabetics as compared to the normal individuals.

## Discussion

Fungi are eukaryotic ubiquitous micro organisms [9] involved in degradation of organic materials [10]. More than 100,000 fungal species were recognized out of that only 150 are pathogenic in nature. In the oral cavity, most frequently isolated species of fungus is *Candida albicans*, a harmless commensal organism.

*Candida* is a dimorphic yeast-like fungus, which can occur in both yeast and hyphae form [11,12]. It is larger than bacteria and smaller than epithelial cells. It is measuring 5-50 μm (length) and 2-5 μm (diameter) in hyphae form and 5-25 μm (diameter) in yeast form [13]. *Candida* replicates asexually by budding yeast form, pseudohyphae form and branching mycelial form. Pseudohyphae form is considered as an invasive phase of *Candida*[14].

*Candida* is a normal commensal of oral mucosa and it can colonize the buccal mucosa, dorsum of the tongue and in denture prosthesis. It can as well adhere to the oral bacteria and pellicle of dental plaque.

Although it is a commensal, it tends to form mild colonization in 20-40% of healthy individuals. In immunocompromised disorders, *Candida* becomes pathogenic [15]; forms moderate to severe colonization and causes oral candidiasis. Oral candidiasis seen more frequently in diabetic patients than in non-diabetics [16,17] and it can be diagnosed from their classical clinical features. However, moderate to severe candidal colonization can occur without any clinical manifestation.

This type of subclinical candidal colonization can make the individual more prone to develop further colonization in other mucosal areas, GIT mucosal region and can also disseminate into the blood circulation [18].

To identify the candidal colonization in diabetic patients, culture methods are available, but these are time consuming. To screen a mass of diabetic patients, we need a simple chair side technique. Oral exfoliative cytology is a very simple, time conserving, effective technique to assess the candidal colonization at the light microscopic level [19,20].

The aim of the present study was to compare the oral candidal colonization between diabetic patients and normal individuals by oral exfoliative cytological method.

In this study, 70% of the normal individuals had mild colonization which denotes their normal commensal nature. But 43.3% of diabetic patients had severe colonization which could be due to their increased tissue glucose level, facilitating the growth of *Candida*.

This study showed that there was no relationship between the degree of oral mucosal candidal colonization in diabetic patients with their age, duration of diabetes, blood sugar level and mode of treatment rendered to them [21].

From this study it was proven that candidal colonization in oral mucosa was higher in diabetics than normal individuals as observed by other studies [22-24]. And also it was proven that even without any clinical lesion of oral candidiasis, diabetic patients tend to have subclinical colonization of opportunistic fungus *Candida albicans*.

## Conclusions

Oral exfoliative cytology is an effective, suitable chair side technique to assess the oral candidal colonization in diabetic patients. This study proved that there was an increased candidal colonization in diabetic patients compared to normal individuals. Early assessment of oral candidal colonization in diabetic patients may help them to avoid colonization in other parts of the mucosal lining of the GIT thereby further dissemination into the blood circulation can be avoided.

## Abbreviations

PAS: Periodic acid schiff; GIT: Gastrointestinal tract.

## Competing interests

There are no competing interests.

## Authors’ contributions

PB and VR designed the study protocol. BPD and NO supervised the study. BP, SI and VG analyzed the grading of candidal colonization. VR and PB wrote the manuscript. Then all authors read and approved the final manuscript.

## Authors’ information

VR. Dean, Professor, H.O.D. Dept of oral pathology and microbiology, Mahatma Gandhi post graduate institute of dental sciences, Pondicherry, India. BPD. Professor, Dept of oral pathology and microbiology, Mahatma Gandhi post graduate institute of dental sciences, Pondicherry, India. NO. Associate professor, Dept of oral pathology and microbiology, Mahatma Gandhi post graduate institute of dental sciences, Pondicherry, India. PB. Junior resident (P.G), Final year M.D.S. Dept of oral pathology and microbiology, Mahatma Gandhi post graduate institute of dental sciences, Pondicherry, India. SI. Junior resident (P.G), Final year M.D.S. Dept of oral pathology and microbiology, Mahatma Gandhi post graduate institute of dental sciences, Pondicherry, India. VG. Junior resident (P.G), Final year M.D.S. Dept of oral pathology and microbiology, Mahatma Gandhi post graduate institute of dental sciences, Pondicherry, India.
